# Time-series analysis of weather and mortality patterns in Nairobi's informal settlements

**DOI:** 10.3402/gha.v5i0.19065

**Published:** 2012-11-23

**Authors:** Thaddaeus Egondi, Catherine Kyobutungi, Sari Kovats, Kanyiva Muindi, Remare Ettarh, Joacim Rocklöv

**Affiliations:** 1 African Population and Health Research Center, Nairobi, Kenya; 2 Department of Public Health and Clinical Medicine, Epidemiology and global Health, Umeå University, Sweden; 3 Department of Social and Environmental Research, London School of Hygiene and Tropical Medicine, London, UK

**Keywords:** time-series, temperature, rainfall, mortality, climate, urban

## Abstract

**Background:**

Many studies have established a link between weather (primarily temperature) and daily mortality in developed countries. However, little is known about this relationship in urban populations in sub-Saharan Africa.

**Objectives:**

The objective of this study was to describe the relationship between daily weather and mortality in Nairobi, Kenya, and to evaluate this relationship with regard to cause of death, age, and sex.

**Methods:**

We utilized mortality data from the Nairobi Urban Health and Demographic Surveillance System and applied time-series models to study the relationship between daily weather and mortality for a population of approximately 60,000 during the period 2003–2008. We used a distributed lag approach to model the delayed effect of weather on mortality, stratified by cause of death, age, and sex.

**Results:**

Increasing temperatures (above 75th percentile) were significantly associated with mortality in children and non-communicable disease (NCD) deaths. We found all-cause mortality of shorter lag of same day and previous day to increase by 3.0% for a 1 degree decrease from the 25th percentile of 18°C (not statistically significant). Mortality among people aged 50+ and children aged below 5 years appeared most susceptible to cold compared to other age groups. Rainfall, in the lag period of 0–29 days, increased all-cause mortality in general, but was found strongest related to mortality among females. Low temperatures were associated with deaths due to acute infections, whereas rainfall was associated with all-cause pneumonia and NCD deaths.

**Conclusions:**

Increases in mortality were associated with both hot and cold weather as well as rainfall in Nairobi, but the relationship differed with regard to age, sex, and cause of death. Our findings indicate that weather-related mortality is a public health concern for the population in the informal settlements of Nairobi, Kenya, especially if current trends in climate change continue.

Climate change arising from human activity has been acknowledged as both an environmental and public health concern. The World Meteorological Organization (WMO) estimates that globally averaged temperatures in 2011 were 0.40°C above the 1961–1990 annual average of 14°C. East Africa was the sixth-warmest region at 1.17°C above normal (1). There is growing evidence that an increase in temperature is associated with short-term increase in morbidity and mortality in several cities across the world (2–6). However, a warmer climate could also correspond to decreases in cold-related mortality, although this has recently been questioned (7). To date, the extent to which cold-related mortality is a problem in East Africa has hardly been explored in the literature. The groups most vulnerable to increasing temperatures are older people and those with pre-existing conditions, such as cardiovascular and respiratory illnesses (3, 5). Some studies have also found that children aged below 5 years are vulnerable as well (8). The effect of rising temperatures is amplified in urban areas where ambient temperatures are compounded by the interaction between air pollution and temperature, the ‘urban heat island effect’ (3), and urban sprawl (9).

The developing world is rapidly urbanizing and estimates show that urban centers will house about 60% of the global population by 2030, with major growth occurring in developing countries. Because of rapid urbanization in periods of poor economic performance, slum settlements are set to increase and population estimates indicate that 41% of global urban population will reside in slums by year 2030 (10). Poor housing characterized by temporary structures is a common feature of slum settlements with most ill-equipped to deal with any adverse events associated with climate change. In addition, increasing urban poverty consigns slum residents to live on the edge with little or no resources to deal with climate-change-related events, including sudden onset of ill health brought on by extreme high or low temperatures (11, 12).

Most studies on temperature-related mortality have been conducted in temperate regions, but little is known about this relationship in developing countries, especially in sub-Saharan Africa. Most sub-Saharan countries lack reliable health outcome data to enable analysis of the impact of weather factors on health. Health Demographic Surveillance Systems (HDSS) provide an opportunity for analyzing the impact of weather on health by providing longitudinal mortality data that allow for time-series analysis. This study investigates:

- the existence of a seasonal pattern in mortality in the Nairobi population,
- the relationship between daily mortality and weather variables (temperature and rainfall), and
- the association between weather and mortality by age, gender, and cause of death.

Overall, this study facilitates a better understanding of weather-related mortality patterns in sub-Saharan Africa and, to the best of our knowledge, the study is the first to explore the weather–mortality linkage for an urban population in Kenya.

## Materials and methods

### Study area and population

The study area covered two informal settlements of Korogocho and Viwandani in Nairobi, which are covered by the Nairobi Urban Health and Demographic Surveillance System (NUHDSS) run by the African Population and Health Research Center. The population under surveillance as on 31 December 2008 was 60,416 individuals from 24,875 households. The study area was characterized by overcrowding, poor sanitation, and poor access to basic health care services. The population in the NUHDSS was mostly youth drawn to the city by the job opportunities (13). The health of the population under surveillance was characterized by high prevalence of infectious diseases and an increasing burden of non-communicable diseases (NCDs). Among the under-five population, diarrheal diseases, perinatal causes, and pneumonia were the leading causes of mortality, whereas HIV and tuberculosis were the major causes of mortality among the population aged five and above (14).

Nairobi city is situated about 1,700 meters above sea level, giving the city a sub-tropical climate. The average daily minimum and maximum temperatures is 11°C and 26°C, respectively. In Nairobi, there are two rainy seasons in a year with the long rainy season from March to May and the short rainy season from October to December. The period from June to August is typically dry and cold, with daily minimum and maximum temperatures reaching 10°C and 21°C, respectively, whereas September, January, and February are hot and dry with daily maximum temperature of 24°C (15, 16).

### Data

Daily mortality data for the study period 2003–2008 was obtained from routinely collected data by the NUHDSS. The analyses were carried out within four age groups: 0–4, 5–19, 20–49, and over 50 years. Age fifty was used as a lower threshold of old age in sub-Saharan Africa based on life expectancy and biomarkers of ageing and the social construction (17, 18). Ascertaining cause of death at NUHDSS was done through the verbal autopsy procedure, which is based on recollections of the deceased person's close relative/caregiver, with the most detailed and credible information regarding the circumstances leading to the death of the individual. Where a close relative or household member cannot be found either due to family relocation after the death or because the deceased lived alone, a credible neighbor is interviewed. Two physicians reviewed verbal autopsy records and when they both agreed on a probable cause of death, it was assigned. If they did not agree, a third physician convened a consensus meeting with the other two, where the disagreement was discussed and if two out of the three agreed on the cause of death, it was assigned. Otherwise, it was designated as indeterminate. Causes of death were classified according to the *International Classification of Diseases*, 10th Revision (ICD-10) using a modified and shortened code list (19). A detailed description of the process is given elsewhere (20). Cause of death was classified into five broad categories: HIV/AIDS related, including TB, (cancer, hypertension, diabetes, and other NCDs), pneumonia, acute infections (meningitis, measles, malaria, and other acute infections) and other natural deaths (perinatal, preterm, and undetermined). For the causes that were determined, HIV/AIDs and TB were the leading causes of mortality with 25.2% of all mortality. The cause of death profile, classified into five groups, is presented in Table 1. Mortality data were broken down to daily series for analysis.

**Table 1:** Cause of death profiles for the study period 2003–2008

Underlying cause of death	*n*	%
**HIV related**		
AIDS	281	11.18
HIV + TB	127	5.05
TB	225	8.95
**Non-communicable**		
Cancers	55	2.19
Diabetes	30	1.19
Hypertension	19	0.76
Other NCDs	209	8.32
**Acute infections**		
Malaria	90	3.58
Measles	61	2.43
Meningitis	77	3.06
Other acute infection	191	7.6
**Pneumonia**	223	8.87
**Other causes**		
Maternal-related death	58	2.31
Malnutrition	50	1.99
Perinatal death	70	2.79
Prematurity/pre-term	29	1.15
Indeterminate	718	28.57

Meteorological data on temperature and rainfall were obtained from the Meteorological Department of Kenya for the period of 2003–2008. The data were obtained from the Moi Airbase weather station, situated between the two study sites and about 3 km away from each site. Weather data included daily minimum and maximum temperatures and daily rainfall. Daily average temperatures were calculated from minimum and maximum temperatures. The weather data were complete, with no missing information for the study period.

### Statistical methods

We used time-series data analysis adapting the Poisson regression model to quantify the relationship between temperature, rainfall, and daily mortality. This approach compared the daily observed and expected mortality based on time trends so as to analyze the deviations from the expected mortality related to variation in the exposure variables, in this case temperature and rainfall. Rainfall was adjusted for temperature effect and vice versa. We investigated the daily weather–mortality relationship allowing for variation in mortality over time by adjusting for season and trend.

To adjust for the influence of seasonal and long-term trends, we included both long and short ‘time’ indicators in the models. The long-term trend was modeled through a natural spline curve with 3 degrees of freedom (df) per year (18 df for 6 years). The annual seasonal variation was also modeled through natural cubic splines with 3 degrees of freedom. Therefore, the combined degrees of freedom for both season and trend added to 6 df per year. Sensitivity analysis was used to assess how the estimates changed for varying degrees of freedom per year (21) to show the sensitivity of results to this choice as described in literature. We examined the relationship between daily deaths and mean temperature to use as baseline analysis. We then assessed the delayed effect through refitting the models using daily mean temperature on the day of death and the previous 13 days using distributed lag models with lag terms 0–1 days, 2–6 days, and 7–13 days. The delayed effect of rainfall over 30 days was assessed considering lag terms of 0–6 days, 7–13 days, 0–13 days, and 14–29 days. Stratified data analysis was conducted with regard to age groups, gender, and cause of death.

Heat and cold temperature thresholds were estimated by visually inspecting the original graph of mean temperature and comparing with different percentiles. The final generalized additive model (GAM) model was given by:

$$Y_{t}\sim \text{Poisson}({µ}_{t})$$

$$\log({µ}_{t})=\alpha +\sum_{i=1}^{3}s(x_{it},df)+s({\text{time}}_{t},df)$$

where *t* refers to the day of the observation; (*Y*_*t*_) denotes the observed daily death counts on day *t*; *s*(.) denotes smooth function; *df* denotes degrees of freedom; *x*_*i*_ denotes the mean temperature at lag 0–1, rainfall at lag 0–13, and rainfall at lag 14–29; and ‘time’ represents both trend and seasonal associations.

To quantify the associations between weather and mortality in different groups of the population, the aforementioned equation was fitted separately for each stratum category of age, gender, and cause. In addition to evaluating the confidence limits and size of coefficients, the relationship was measured by a factor of 1°C for temperature and 1 inch (25 mm) for rainfall. All data management was conducted in STATA version 11 and statistical analyses were conducted using the *mgcv* package in R2.14.2. Quasi-Poisson family was used instead of Poisson for adjustment of over-dispersion that may partly result because of many zero counts. Quasi-Poisson does not change the estimates for the coefficients, but adjusts the standard errors to account for over-dispersion.

## Results

### Description

The distribution of mortality by cause, sex, and age for the period of 2003 to 2008 is presented in Table 2. There were 2,512 non-accidental deaths during the study period (excluding deaths due to injuries). The proportion of deaths among children younger than five years was 33.7%, whereas older persons (50+ years) accounted for 13.9% of deaths. People aged between 5 and 19 accounted for about 5% of the deaths and because of this small proportion, it was combined with deaths among people aged between 20 and 49 (whose proportion was 47.2%).

**Table 2:** Distribution of mortality by age, gender, and cause of death

	*n*	Percent	Daily average	Daily maximum
**Age**				
All ages	2,512	100	1.19	13
0–4 years	847	33.7	0.40	4
5–19 years	131	5.2	0.06	2
20–49 years	1,186	47.2	0.56	10
50+ years	348	13.9	0.16	4
**Sex**				
Female	1,180	47.0	0.56	7
Male	1,332	53.0	0.63	9
**Cause of death**				
HIV	632	25.2	0.30	5
NCDs	313	12.5	0.15	4
Pneumonia	223	8.9	0.11	3
Acute infections	419	16.7	0.20	4
Other causes	925	36.8	0.44	9

Summary statistics for weather variables over the study period indicate that the average daily mean temperature was 18.8°C (SD = 1.7). The average daily minimum temperature was 13.5°C and the average daily maximum temperature was 23.4°C. During this period, the lowest temperature experienced was 5.2°C and the maximum was 30.7°. The daily average temperature range was 10.3°C with the highest difference of 19.9°C. The highest amount of rainfall was 99.7 mm observed in the year 2007 and on rain-days the average amount of rainfall was 8.2 mm.

### Seasonal variability of weather and mortality

**Fig. 1:**
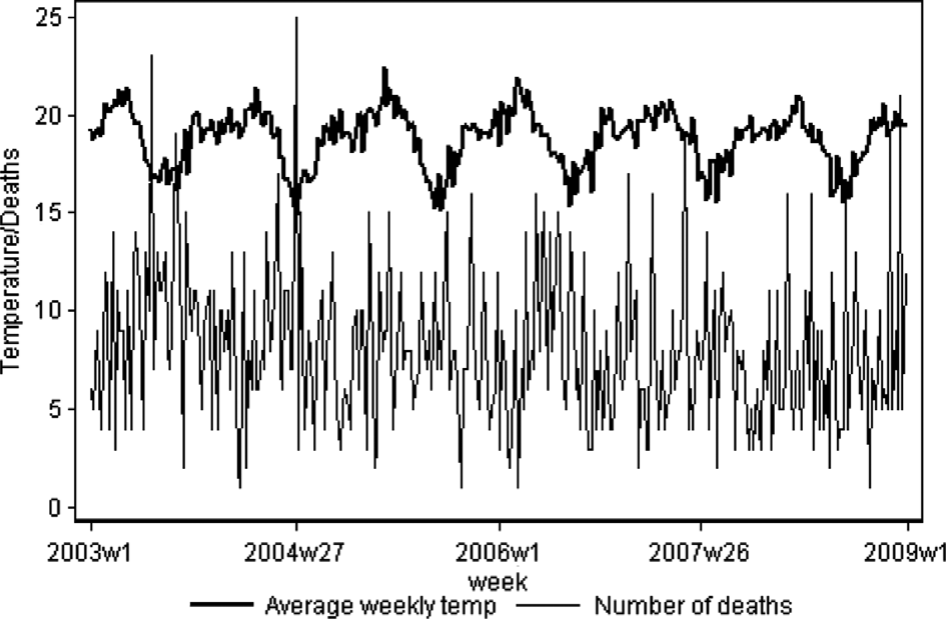
Time series of all-cause (weekly) mortality and temperature (°C).

Seasonal variation in all-cause mortality and mean temperature for the period 2003–2008 is shown in Fig. 1. Overall, there are seasonal fluctuations in mortality, with the highest rates of death occurring during periods of relative cold, which coincides with high amounts of rainfall. In contrast to the broadly observed association between cold periods and mortality, no seasonal increase in mortality was clearly discernible from this graph during periods of highest temperature.

**Fig. 2:**
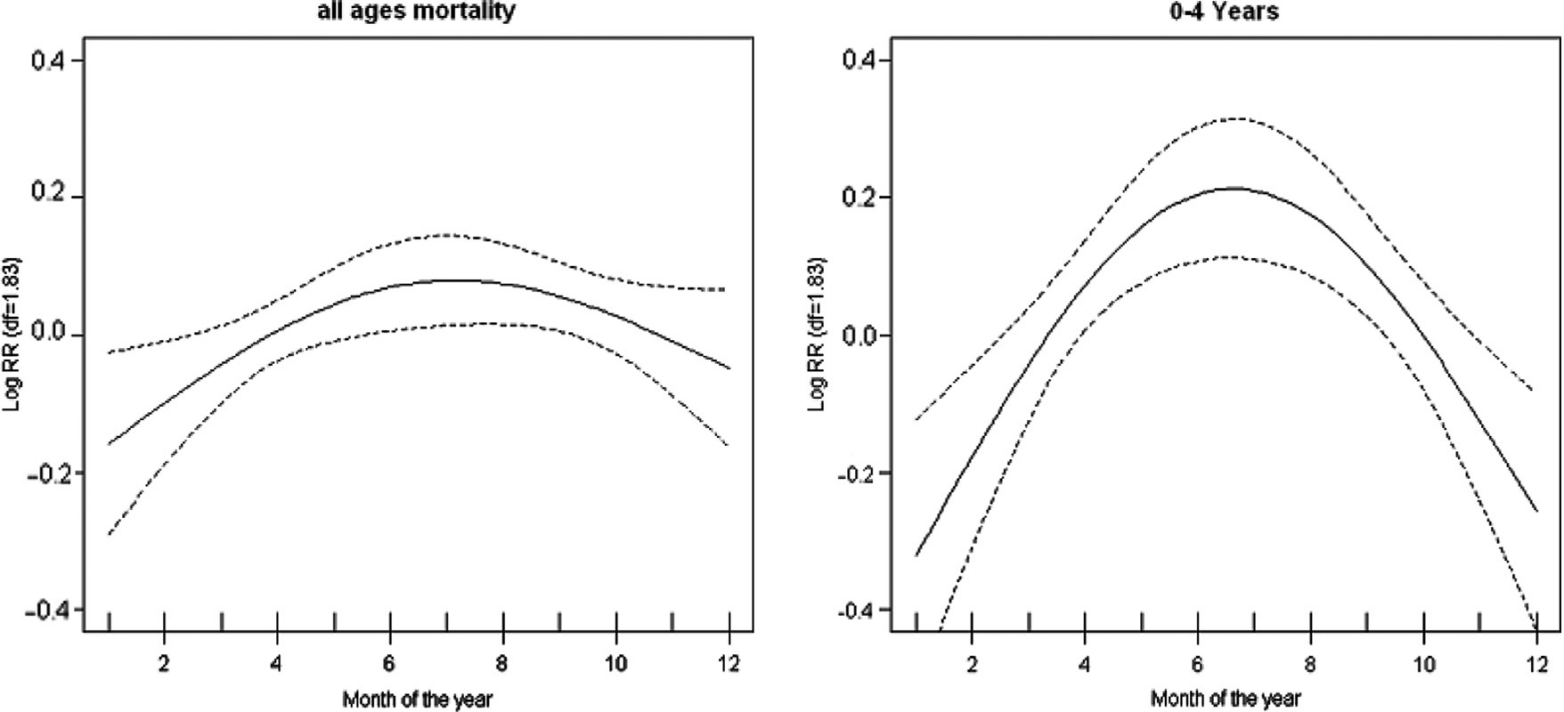
Annual seasonal variation plots for all-age and under-five mortality. The vertical axes show the log (relative risk) and the horizontal axis show the month starting with January. Confidence intervals (95%) are shown as dotted lines.

There was no strong seasonal pattern observed for mortality among the ages 5–49 and 50+ years except for under-five mortality. The two plots for seasonal mortality, all-age and under-five, are shown in Fig. 2. We observed a similar pattern for the two plots, but a strong seasonal pattern is observed for under-five mortality. This result implies that the seasonal pattern observed for all-age mortality was driven mainly by the under-five deaths. High mortality was observed during the month of June to July, the period corresponding to low temperatures. The plots show that mortality risk over the year rises from the lowest mortality risk by about 40% in the 0–4 age group and by about 20% for all ages. These estimates are obtained by exponentiation of the values of log relative risk shown in Fig. 2.

### Temperature and rainfall mortality plots

**Fig. 3:**
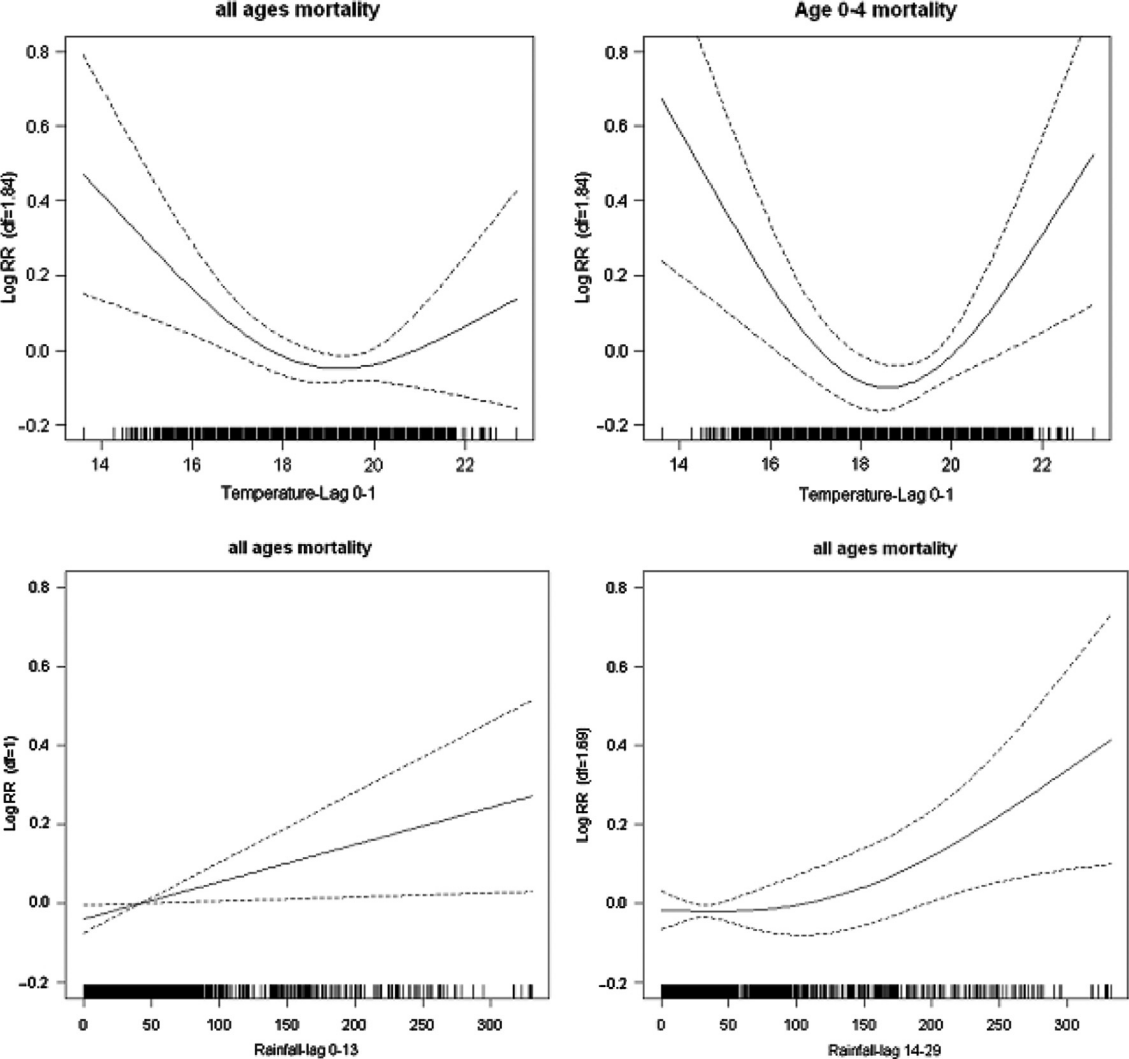
Smooth functions of temperature for all and under-five mortality, and rainfall for all ages allowing lags of 0–13 days and 14–29 days. The vertical axes show the log (relative risk) and the horizontal axis show the scale of the explanatory variable. Confidence intervals (95%) are shown as dotted lines.

The graphs in Fig. 3 show smoothed plots of log relative risk of mortality against the mean of the current and previous day's temperature. The graphs reveal non-linear temperature–mortality relationships. From the figure, it is evident that a threshold temperature exists somewhere between 18°C and 20°C for all-age mortality. These thresholds from the initial graphical inspection revealed cold and heat threshold corresponding to 25th and 75th percentiles, respectively. The actual daily average temperatures corresponding to 25th and 75th percentiles were 17.9°C and 20°C, respectively. These thresholds were used for all subsequent analysis for quantifying the relationship between temperature and mortality as linear functions. The pattern of temperature and mortality association exhibits J-shape for all-ages mortality and U-shape for under-five mortality. The negative association was observed for the temperature range below the 25th percentile threshold of 17.9°C, whereas a positive association existed for the temperature range above the 75th percentile threshold of 20°C. The slopes for under-five were steeper than for all mortality but both non-linear relationships were statistically significant at lag 0–1. This relationship implies that decrease or increase from the average daily temperature was associated with increase in mortality. The smooth function of the relationship between rainfall and mortality shows a linear increase, significant at lag of 0–13 days and 14–29 days for all-cause mortality. These associations are also illustrated in Fig. 3.

### Quantification of weather–mortality relationships

Table 3 shows the results of a quantified relationship varied by age, gender, and cause of death. The results show no significant relationship for high temperature on all mortality, but significant positive relationship for high temperatures is observed in deaths in the 0–4 age group and among people with NCD. Low temperatures were associated with mortality of varying magnitude by age, gender, and cause of death though none of the relationships was statistically significant. There was an increase of 13% in deaths due to acute infections associated with decrease in temperature but this was not statistically significant. Deaths among males and people aged 50+ were associated with low temperatures though these relationships were not statistically significant. It was also observed that rainfall was significantly associated with female NCDs and pneumonia deaths. Cumulatively, mortality increased by 3% for 1 inch (25 mm) increase in the amount of rainfall and this relationship was statistically significant. There was also an increase of 5% in female mortality for 1 inch (25 mm) increase in rainfall after 2 weeks and this increase was statistically significant. The relationship of rainfall to NCDs and pneumonia accumulated over 1 month was 12% and 24%, respectively, and this was statistically significant.

**Table 3:** Percentage change associated with 1°C decrease in temperature below 25th percentile, 1°C increase in temperature above 75th percentile and 25.4 mm (1 inch) increase in amount of rainfall. 95% confidence intervals are given in the parentheses

	Temperature		Rainfall		
		
	25th percentile	75th percentile	Lag 0–13 days	Lag 14–29 days	Cumulative
**All deaths**	3 (−5, 13)	0 (−1, 1)	2 (−1, 5)	1 (−1, 4)	3 (0, 7)
**Age groups**					
0–4 years	3 (−9, 16)	1 (0, 2)	2 (−2, 6)	0 (−3, 4)	2 (3, 8)
5–49 years	2 (−8, 14)	0 (−1, 1)	2 (−1, 6)	1 (−2, 4)	3 (−1, 8)
50+ years	9 (−6, 28)	1 (−1, 2)	3 (−2, 8)	3 (−2, 7)	5 (−1, 13)
**Gender**					
Female	−3 (−8, 13)	0 (−1, 2)	5 (1, 8)	2 (−1, 6)	7 (2, 12)
Male	10 (−1, 22)	0 (−1, 1)	0 (−3, 3)	0 (−3, 4)	0 (−4, 5)
**Cause of death**					
HIV	2 (−11, 17)	0 (−2, 1)	1 (−3, 5)	−1 (−5, 3)	0 (−5, 6)
NCDs	−9 (−22, 6)	1 (0, 3)	6 (1, 12)	6 (1, 11)	12 (5, 20)
Pneumonia	6 (−21, 11)	1 (−1, 2)	13 (7, 19)	10 (5, 15)	24 (16, 33)
Acute	13 (−2, 30)	1 (−1, 2)	−2 (−7, 2)	0 (−4, 4)	−2 (−8, 4)
Other	1 (−11, 14)	0 (−1, 2)	2 (−2, 6)	1 (−3, 4)	2 (−3, 8)

### Sensitivity analysis

**Fig. 4:**
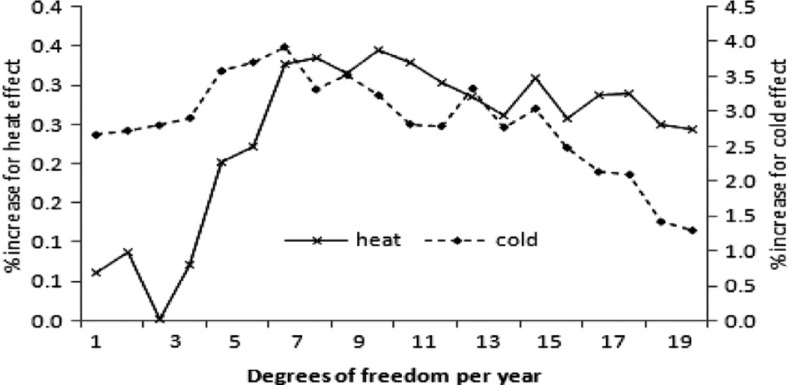
Sensitivity analyses, with increasing degrees of freedom, of the percentage increase in mortality for an increase in temperature of 1°C at lag 0–1 days for both cold and heat effects. The left vertical axis shows the heat effect and right vertical axis shows cold effect in percentages.

Figure 4 illustrates a sensitivity analysis of the percentage increase in mortality for a decrease or an increase in temperature of 1°C at lag 0–1 for both cold and heat effects with respect to the number of degrees of freedom assigned to the smooth function of time per year. The figure shows the change in the temperature-associated coefficient as the degree of freedom is varied. The different axes are used for cold and heat correlations because of the difference in the magnitude of the effect on the percentage change on mortality. When using natural splines, the estimates reach the maximum when degree of freedom is about six per year. This implies that a choice of degrees of freedom lower or higher than this optimal value will lead to over- or under-estimation of the effect of weather variables.

## Discussion

The aim of this study was to assess the relationship between daily weather and mortality in two informal settlements in Nairobi, Kenya. The findings show a relationship between both low and high temperature with mortality. The observed linear associations between temperatures below 25th percentile were not statistically significant. Increase in temperature above the 75th percentile showed statistically significant but moderate increases in mortality in children/infants and for NCDs. The lack of significance for the relationship between cold temperature and mortality may be due to low daily death counts resulting in high variability or due to the particular thresholds chosen since some of the non-linear curves were significant. The results also show strong statistically significant positive relationships between rainfall and mortality, with cumulative lagged effect over 30 days in the following groups: all ages, NCDs, and pneumonia.

The temperature-related mortality was observed with a short lag of the same day and the following day. The low temperature effect could be explained by the high elevation of the city, resulting in a sub-tropical (almost temperate) climate, which is cool almost all year round, with no temperature extremes reported so far. It is plausible therefore that a cold effect can be found in this kind of climate where temperature extremes on the cold scale have been reported, especially at nighttime, and housing lacks insulation or proper heating, with natural heating fuels being limited or relatively expensive. The low temperatures may be associated with use of various fuels to generate warmth increasing exposure to indoor pollution. Commonly used fuel types by the residents in the two study areas are kerosene and charcoal, which are the main sources of indoor air pollutants. This may be a probable explanation for the association of low temperatures with mortality. Our results are in line with previous findings assessing weather-related mortality. Other studies in temperate countries have reported increased mortality associated with cold waves (22, 23). Although the temperatures in Nairobi are not fully comparable to those in the studies above, a parallel can be drawn in the increased mortality observed during cold days. Studies of temperature-related mortality in Bangladesh observed an increase in all-cause deaths at low temperatures and no clear heat effects in both rural and urban areas (24, 25).

Earlier studies focused on the effect of short temperature extremes, such as heat waves (26). Temperature-related mortality typically demonstrates a J or U shaped response, in which mortality rates are highest at low and high temperatures. All-cause mortality is associated with a decrease in temperature from a cold threshold (27–30), and in this study low-temperature-related mortality was higher than high-temperature-related mortality though it was not statistically significant. The effects of low temperatures on mortality can last for days, with the greatest association sometimes observed on the same day (31) as observed in this study. Infectious diseases such as pneumonia and influenza are more common in the winter season in temperate countries and contribute to the observed high rates of winter mortality (32–34). In contrast, we found that pneumonia deaths increased with high temperatures and vice versa. There was no information about influenza in this area. Respiratory tract infections are more likely to occur during periods of low temperatures and low humidity (35).

We also observed an association between rainfall and mortality, particularly with pneumonia deaths. Previous work showed an association between rainfall and pediatric visits for acute gastrointestinal illness (36). In addressing climate change, it is better to understand the rainfall-associated illness or mortality. Establishing the impact of rainfall on health in the absence of any outbreaks is important in public health reporting, not to underestimate rainfall-associated mortality or illness. Further studies on impact of rainfall are warranted to better understand this association and potential mechanisms as well as establish different lag effects of rainfall.

Older populations are considered particularly vulnerable to extreme weather because a person's ability to thermo-regulate can become impaired with age. Underlying chronic diseases, such as diabetes, and medications can modify blood pressure, circulation, perspiration rates, and some mental capacities such as warmth perception, thus complicating people's ability to identify when they are experiencing extreme weather (37). Several reports have shown that excess weather-related mortality is higher in older people (22, 38, 39). Infants are also often identified as a population that is vulnerable to extreme heat conditions; however, information on heat and infant mortality is scarce, with no studies reporting on cause-specific temperature-related mortality in children (8). In an effort to address Millennium Development Goal (MDG) 4, it is important to confirm weather-related mortality among children to understand factors contributing to child mortality. The strong association observed between temperature and mortality among children aged 0–4 years show the need of further cause-specific analysis for this age group.

Gender has been found to be associated with temperature-related mortality. Some studies have shown that women are more sensitive to cold than men, with reports showing that women living in colder climates have a higher risk of cold-related death than their male counterparts (27, 40, 41). We found increased cold-related mortality among men although previous work shows contrasting results. However, this could be explained by the difference in the age, culture, and behavior of the population under study. A similar finding was observed in a study among older people (42) though a contradicting result was also observed in a different study among older people of a similar age group (43). Little modification of the cold effect by sex was observed in England and Wales (44). We further observed a significant relationship between increasing rainfall and mortality in women, but not in men. The reason for this relationship needs to be further assessed.

In spite of an overall trend toward increasing global temperatures, climate models forecast more variable weather. This will result in important weather-related health consequences for humans. Coupling archived climatologic data with health outcome data has aided researchers (45) in projecting mortality rates and in this study the NUHDSS provides this opportunity. Further epidemiologic studies that incorporate archived climatological and environmental data in modeling specific health outcomes in vulnerable populations would aid adaptation to climate-change-related health effects through preparedness strategies implemented at various scales (37). Although the world will get warmer in the future, the low temperature-related mortality is likely to remain an important concern (7).

This study has a number of limitations. The first limitation relates to the validity of the causes of death based on verbal autopsy. However, verbal autopsies are at present the best possible method for obtaining information on cause-specific deaths in many low- and middle-income countries (46) that lack vital registration systems and where most deaths occur outside the formal health care system. Another limitation with mortality data is the large percentage of deaths with unknown or ill-defined causes, making cause-specific mortality analysis difficult. Nevertheless, the data provide a great opportunity for all-cause mortality analysis. A further limitation is the lack of data on daily levels of air pollution, which would enable the control of its health impact while assessing weather-related mortality. Low death counts may be also a limitation, resulting in large variability that renders the observed differences as not significant, although this has no significant effect on the Poisson distribution of the model we used. The adjustment to standard errors is done through quasi-likelihood estimation. Despite these limitations, the study confirms that extremes of temperature affect mortality in the Nairobi Urban HDSS and highlights seasonal variations in mortality. Further investigations of weather-related mortality are warranted.

In conclusion, we found the highest susceptibility to heat among children younger than five years and an indication of cold-related mortality among the older people. Results from stratifying effect estimates by age were consistent with earlier results based on community-level and individual-level data (47–49). Further investigation using individual-level data is needed to improve exposure estimates, especially for longer lag structures. These findings on the impact of weather on mortality have implications for policymakers and health protection for vulnerable urban populations to weather extremes. The identified susceptible subpopulations signify the need for targeted weather-mortality prevention efforts such as proper housing and clothing.
